# Treatise on Diseases and Injuries of the Eye

**Published:** 1874-01

**Authors:** 


*Treatise on the Diseases and Injuries of the Eye, Including the Anatomy of the Organ.* By Dr. Carl Stellwag. Translated from the fourth German Edition. By D. B. St. John Roosa, M. D., Charles S. Bull, M. D., and Charles E. Hackley, M D., New York: Wm. Wood & Co., 1873. Buffalo: H. H. Otis.

The present edition of the American Translation of Prof. Stellwag's Treatise, is presented in a form thoroughly revised and considerably enlarged. Over one hundred new pages have been added, and the whole work bears evidence of a thorough revision of its contents. Having passed through three previous American Editions, it is so well known to the profession that any thing but a notice of its republication is unnecessary. In the additions which have been made, is a description of two new methods of extracting Cataract. The translators have also included in the form of an appendix the subject of direct examination of the fundus of the eye, in which is included an illustrated description of Dr. Loring's adaptation of the ophthalmoscope to this method.

Replete with all that is desirable in a complete treatise on the subject of Ophthalmology, this fourth Edition of Prof. Stellwag's work is one which may be studied by all with profit. When the student has mastered its contents he may be said to have truly laid the foundation for a knowledge of Ophthalmology, which, if properly built upon will place him in full possession of all that is known upon the subject. While the author expresses his own opinions, founded upon extensive and careful observation, in a forcible manner he has nevertheless a just respect for the views of other laborers in the same field, and gives his readers the full value of their observations. In doing this he does not enter into a long and tiresome debate concerning the merits or demerits of certain views but after expressing plainly his own opinion leaves his reader to decide for himself. By those who have not had the benefit of a German Education, this English Edition of his work will be highly prized.

